# Expression Profile and Regulatory Properties of m6A-Modified circRNAs in the Longissimus Dorsi of Queshan Black and Large White Pigs

**DOI:** 10.3390/ani13132190

**Published:** 2023-07-04

**Authors:** Kunlong Qi, Yaqing Dou, Zhe Zhang, Yilin Wei, Chenglei Song, Ruimin Qiao, Xiuling Li, Feng Yang, Kejun Wang, Xinjian Li, Xuelei Han

**Affiliations:** College of Animal Science and Technology, Henan Agricultural University, Zhengzhou 450046, China

**Keywords:** N6-methyladenosine, Queshan Black pig, circRNA, fat deposition, meat quality

## Abstract

**Simple Summary:**

With the improvement of living standards, people have higher requirements for pork quality. Intramuscular fat is among the important factors that determine the quality of pork, affecting the taste and flavor. Producing quality meat with high intramuscular fat content has been a major challenge for China’s livestock industry. In addition, m6A modification is abundant in many circRNAs, and m6A-circRNAs play an important regulatory role in a variety of biological processes. In this study, a total of 12 putative m6A-circRNAs were identified, their potential molecular regulatory mechanisms on fat deposition and meat quality in Chinese and Western pigs were explored through a series of bioinformatics analyses, which laid a foundation for further elucidations of the functions of m6A-circRNAs in fat deposition and growth and development of pigs.

**Abstract:**

It is well known that N6-methyladenosine (m6A) is the most abundant modification in linear RNA molecules, but many circRNA molecules have now been found to have a wide range of m6A modification sites as well. However, there are few relevant studies and information on the expression profile and functional regulatory properties of m6A-modified circRNAs (m6A-circRNAs) in longissimus dorsi. In this study, a total of 12 putative m6A-circRNAs were identified and characterized in the longissimus dorsi of Queshan Black and Large White pigs—8 of them were significantly more expressed in the longissimus dorsi of Queshan Black than in Large White pigs, while the other 4 were the opposite. These 12 putative m6A-circRNAs were also found to act as miRNA sponge molecules to regulate fat deposition by constructing the ceRNA regulatory network. Enrichment analysis also revealed that the 12 m6A-circRNAs parent genes and their adsorbed miRNA target genes were widely involved in fat deposition and cell proliferation and differentiation-related pathways, such as the HIF-1 signaling pathway, the pentose phosphate pathway, the MAPK signaling pathway, the glycosphingolipid biosynthesis-lacto and neolacto series, and the TNF signaling pathway, suggesting that the analyzed m6A-circRNAs may be largely involved in the formation of pork quality. These results provide new information to study the regulatory properties of m6A-circRNAs in the formation of pork quality.

## 1. Introduction

Pork is an important source of animal protein and due to the improvement in living standards of people in recent years, the quality of pork has become more important than the quantity, but there are more factors affecting the quality of pork, mainly including intramuscular fat content, drip loss, tenderness, shear force, and marbling [1]. Queshan Black pig is a domestic breed with a long history and is a typical fat pig with high intramuscular fat content and excellent meat quality. Therefore, it is especially important to explore the markers that affect the quality of Queshan Black pig pork. Furthermore, it is worth noting that pigs can be used as animal models to study some diseases because they are anatomically and physiologically similar to humans [2]. Understanding the mechanisms of fat deposition in pig muscle can help reveal the molecular mechanisms affecting pork quality, and it is necessary to discover and characterize new regulatory factors associated with influencing pork quality.

It has been more than 40 years since the discovery of circRNAs, which are known to be a class of biologically active nucleic acid molecules that can form covalently closed continuous loops of ncRNAs without 5′-3′ polarity and polyadenylated tails, produced by being back-spliced [3]. Most circRNAs are conserved among species and are resistant to exoribonuclease RNase R, so they are more stable than linear RNAs due to their loop structure [4,5]. CircRNAs are usually classified as noncoding RNAs (ncRNAs), although some circRNAs have protein-coding potential [6,7,8]. CircRNAs can be mainly classified into exonic circular RNAs, intronic circular RNAs, and intergenic circular RNAs or circRNAs derived from long noncoding RNA. There is increasing evidence that circRNAs play a key role in regulating meat quality. It has been found that circMARK3 promotes adipogenic differentiation of buffalo adipocytes and 3T3-L1 cells by upregulating the expression level of adipogenic marker genes, which are potential factors for promoting adipose deposition by regulating the differentiation and adipogenesis of buffalo adipocyte [9]. Analysis of circRNA in the longissimus dorsi muscle of Huainan and Large White pigs at three stages revealed that circ_0030593 and circ_0032760 might be involved in intramuscular fat deposition [10]. CircSETBP1 was found to be a newly identified circRNA associated with adipogenesis, it controlled the proliferation and differentiation of porcine intramuscular fat preadipocytes and 3T3-L1 cells by regulating the miR-149-5p/CRTCs axis [11].

RNA modifications include methylation, hydroxymethylation, and acetylation, while N6-methyladenosine (m6A) is the most abundant modification of RNA, accounting for 80% of its methylation modifications, with an average of one to three m6A modifications per transcript [12,13]. M6A methylation refers to the chemical modification phenomenon in which methyl-adenine of RNA is selectively added with methyl groups under the catalysis of methyltransferase. It is the most common modification on higher biological mRNAs and long noncoding RNA. However, with the increasing number of studies, it was gradually found that m6A modification can also occur on circRNA. To date, an increasing number of studies have found that m6A-modified circRNAs play important roles in different biological processes. The m6A modification of circHPS5 was found to regulate cytoplasmic export and increase HMGA2 expression to promote the development of hepatocellular carcinoma [14]. Upregulation of circCCDC134 with m6A modifications in cervical cancer was found to be fine-tuned by ALKBH5-mediated m6A modifications to enhance its stability in a YTHDF2-dependent manner. CircCCDC134 enhanced tumor proliferation and metastasis in vitro and in vivo [15]. The study of secondary hair follicle stem cells revealed that circRNA-ZNF638 promotes the induced activation of secondary hair follicle stem cells in an m6A-dependent manner through the miR-361-5p/Wnt5a axis in cashmere goats [16].

In this study, based on whole transcriptome sequencing and analysis of the longissimus dorsi from different breeds (Queshan Black and Large White pigs) from both domestic and foreign sources, we identified 62 differentially expressed circRNAs, and predicted the methylation modification of these circRNAs, resulting in 12 putative m6A-methylated circRNAs. Of these, four m6A-circRNAs were found to be lower expressed in Queshan Black than in Large White pigs, whereas the expression of the remaining eight was reversed, in other words, higher expressed in Queshan Black than in Large White pigs. Furthermore, in this study, we analyzed these 12 putative m6A-circRNAs by bioinformatics and generated a regulatory network of competing endogenous RNAs (ceRNA) by predicting their sponge miRNAs and target genes, and, at the same time, performed pathway enrichment analysis of their parent genes and potential regulatory genes. Finally, putative expression patterns of m6A-circRNAs with the corresponding parent genes were characterized in Queshan Black and Large White pigs. These results will pave the way for elucidating the biological functions and regulatory features of m6A-circRNAs in intramuscular fat deposition.

## 2. Materials and Methods

### 2.1. Sample Collection, Sequencing, and m6A-circRNA Prediction Analysis

Sample collection was described previously. Briefly, the experimental animals were three Queshan Black and three Large White pigs, all of which were castrated boars in this study, and samples were frozen in liquid nitrogen and stored at −80 °C until RNA extraction [17,18]. Total RNA was extracted from the samples using TRIzol reagent (15596026, Thermo Fisher Scientific, Waltham, MA, USA), and its concentration and quality were tested for satisfactory sequencing analysis. Using this as a basis, this study further used the SRAMP program (http://www.cuilab.cn/sramp, accessed on 2 January 2023) to predict the putative m6A sites in the sequences of the 64 differentially expressed circRNAs obtained in the Queshan Black and Large White pigs [18], and selected at least two m6A sites containing very high confidence as putative m6A-circRNAs. The standards and parameters used to determine the confidence level of identified m6A sites in circRNA correspond to very high/high/moderate/low confidence levels. The thresholds for very high/high/moderate/low confidence m6A sites correspond to the thresholds achieved, 99%/95%/90%/85% specificities on cross-validation tests, respectively.

### 2.2. Putative m6A-circRNA Sequence Analysis, Open Reading Frame (ORF) and Internal Ribosome Entry Site (IRES) Prediction

The m6A-circRNAs analyzed in this study were identified based on our previous identification of circRNAs in the longissimus dorsi muscle transcriptomes of the Queshan Black and Large White pigs [18]. The length, chromosome distribution, and sequencing results of these circRNAs were also analyzed. Importantly, their nucleotide composition and frequency distribution were statistically analyzed using an online tool (http://www.detaibio.com/sms2/dna_stats.html, accessed on 1 February 2023) [19]. The open reading frame (ORF) of each transcript was identified using cirPrimer software (version 2.0) [20]. The IRES is an important regulatory element that allows RNA to be translated independently of the cap structure at the 5’ end. IRES-mediated protein translation also plays a role in physiological or pathological processes such as cellular stress response, particularly in circRNAs, where it mediates the assembly of ribosomal subunits and ultimately results in the translation of circRNAs into polypeptides or proteins [21]. We used the IRESfinder software to predict whether circRNA sequences have potential IRES elements. IRESfinder is a novel logit model trained on experimentally validated IRESs, and has a high predictive efficiency for identifying IRESs in the spliced sequences of identified circRNAs [22].

### 2.3. Functional Enrichment Analysis of m6A-circRNA Parent and Target Genes

To preliminarily identify the function of 12 m6A-circRNAs, this study performed functional enrichment analysis of their parent and target genes. In order to perform functional enrichment analysis of its target genes, the final target gene prediction results were first crossed by miRanda (version 3.3a) [23], PITA [24] and RNAhybrid (version 2.2) [25] software, and Gene Ontology (GO) and Kyoto Encyclopedia of Genes and Genomes (KEGG) enrichment analysis were performed. GO is an internationally standardized gene function classification system that provides a dynamically updated controlled vocabulary to fully describe the properties of genes and gene products in organisms [26]. KEGG is the main public database for the pathway. The pathway significant enrichment analysis used the KEGG pathway as a unit, and applied a hypergeometric test to find out the pathway that was significantly enriched in differentially expressed genes compared to the entire genome background [27]. GO and KEGG analysis was performed using the OmicShare tools, an online platform for data analysis (https://www.omicshare.com/tools, accessed on 31 January 2023).

### 2.4. Construction of the m6A-circRNA–miRNA–mRNA Network

m6A-circRNA can act as a sponge by binding to the “seed” region of miRNAs [11]. The miRNA binding sites of the spliced putative m6A-circRNA were predicted using miRanda and TargetFinder [28] software. The m6A-circRNA–miRNA–mRNA network was constructed by combining all the above identified co-expression competition triplets, and subsequently visualized by Cytoscape (version 3.9.1, http://cytoscape.org/, accessed on 9 January 2023) [29].

### 2.5. m6A RNA Methylation Quantification (Me-RIP)

After extraction, the total RNA was treated with RNase R (Cat. No. R0301, GENESEED, Guangzhou, China) and then fragmented using the RNA Fragmentation Buffer in the m6A Methylated RNA Immunoprecipitation Kit (C11051-1, RiboBio Biotechnology, Guangzhou, China), and 1/10th of the product was stored as an input control at −80 °C. The remaining RNA was incubated for 2 h at 4 °C in a rotary mixer with prepared magnetic beads having 5 µg of anti-m6A antibody. Next, elution and RNA recovery were performed by adding 100 μL of elution buffer and shaking at 4 °C for 1 h. After transferring to new centrifuge tubes, the RNA was recovered using the Hipure Serum/Plasma miRNA Kit (R4318-01, Magen Biotechnology, Guangzhou, China) in order to obtain the IP-group RNA captured by the m6A antibody, and finally the expression level of m6A-circRNA was detected by qRT-PCR.

### 2.6. MeRIP-qPCR

RNA extracted from muscle was reverse-transcribed into cDNA using the Evo M-MLV RT Kit with gDNA Clean for qPCR (Code No. AG11705, Accurate Biotechnology, Changsha, Hunan, China) to convert total RNA to cDNA according to the manufacturer’s instructions. Next, qRT-PCR was performed using SYBR^®^ Green Premix Pro Taq HS qPCR Kit (Code No. AG11701, Accurate Biotechnology, Changsha, Hunan, China) on the CFX96 Real Time PCR Detection System (Bio-Rad, Hercules, CA, USA). The reaction program was as follows: denaturation at 95 °C for 3 min, followed by 45 cycles at 95 °C for 10 s and 60 °C for 30 s. MeRIP-qPCR does not require internal reference genes, and the IP/input ratio is calculated by 2^−ΔCt^ (ΔCt = Ct_IP_ − Ct_input_) and the ratio of IP RNA template and input RNA template to initial RNA when reverse transcription is introduced. All primer sequences are listed in Table 1.

### 2.7. Statistical Analysis

R (version 4.2.2, https://www.r-project.org/, accessed on 9 January 2023) was used for statistical analysis as well as for data visualization. Based on the RNA-seq results, to demonstrate the expression of the 12 putative m6A-circRNAs in Queshan Black (*n* = 3) and Large White pigs (*n* = 3), heat map was performed using the OmicShare tools, an online platform for data analysis (https://www.omicshare.com/tools, accessed on 4 February 2023). The data obtained from this study were presented as the mean ± SD. Differences among groups were analyzed using Student’s t-test. *p* < 0.05 was regarded as statistically significant.

## 3. Results

### 3.1. Identification and Characterization of m6A-circRNAs in the Longissimus Dorsi of Queshan Black and Large White Pigs

**Figure 1:**
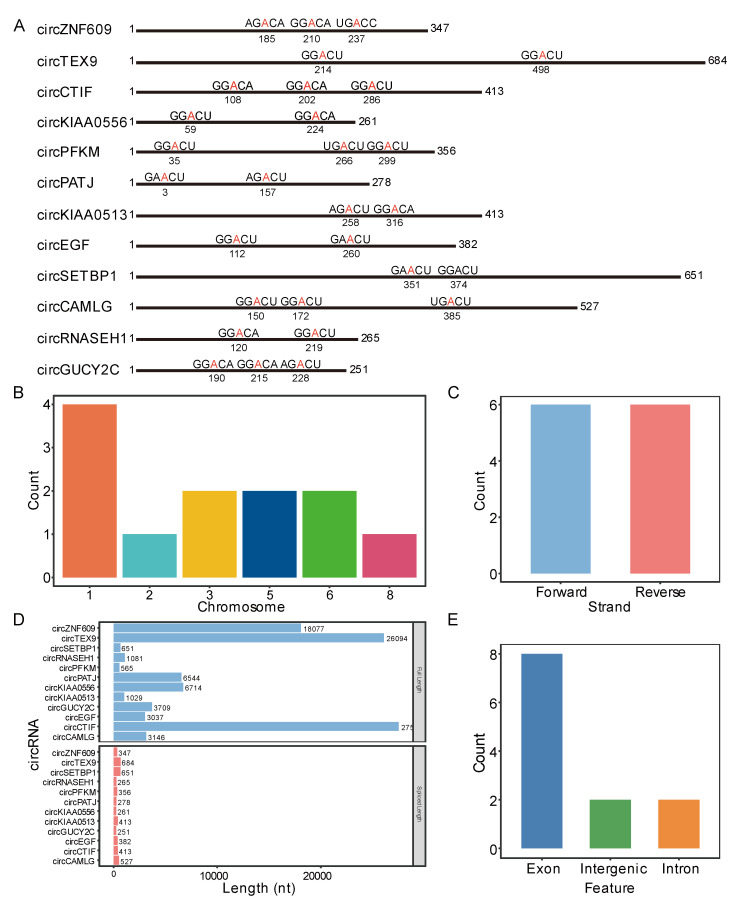
Identification and characterization of m6A-circRNAs in the longissimus dorsi of Queshan Black and Large White pigs. (**A**) Schematic diagram of the putative m6A sites in the 12 circRNA sequences in the longissimus dorsi of the Queshan Black and the Large White pigs. The black lines represent the circRNA sequences, and the numbers below the black lines represent the corresponding nucleotide positions of the m6A sites in the circRNA sequences. (**B**) The distribution of 12 putative m6A-circRNAs on different chromosomes, (**C**) the distribution of chromosomal forward and reverse strands, (**D**) the length changes before and after being spliced, and (**E**) their source regions.

There have been an increasing number of studies that have so far shown that many circRNAs contain m6A modification sites and play an important role in various life activities. Therefore, in this study, using SRAMP analysis, we screened m6A-circRNAs from Queshan Black and Large White pigs, and obtained a total of 12 putative m6A-circRNAs with very high confidence m6A sites, and it was observed that each motif of the m6A sites in these circRNA sequences is identical to the motif of the m6A site found in the linear RNA molecule: RRACH (R: A/G; H: A/C/U) (Figure 1A). In addition, putative m6A-circRNAs were also found to map to different chromosomes, but those located on chromosome 1 were predominant (Figure 1B), while these putative m6A-circRNAs were evenly distributed on the forward and reverse strands of chromosomes (Figure 1C).

In this study, the characteristics of 12 m6A-circRNAs were analyzed and the results showed that the tail end of the last exon was connected to the front end of the first exon through specific back splicing, without the 5’ short cap structure and 3’ polyA tail, resulting in a significant change in their length before and after being spliced (Figure 1D). In addition, most putative m6A-circRNAs were found to be formed by exon cyclization, followed by intergenic regions and introns (Figure 1E). Exonic circRNA is generated by exon skipping and then forms a circular structure by back splicing. Introns rely on 5 ‘and 3’ motif (AG-GU) back splicing to drive cyclization and then shear to form mature intronic circRNA. Exon-intron circRNA contains both exons and introns. An intergenic region is a sequence of intervals between each gene that does not belong to exons or introns.

### 3.2. Sequence Analysis and Coding Ability Prediction of Putative m6A-circRNAs

**Figure 2:**
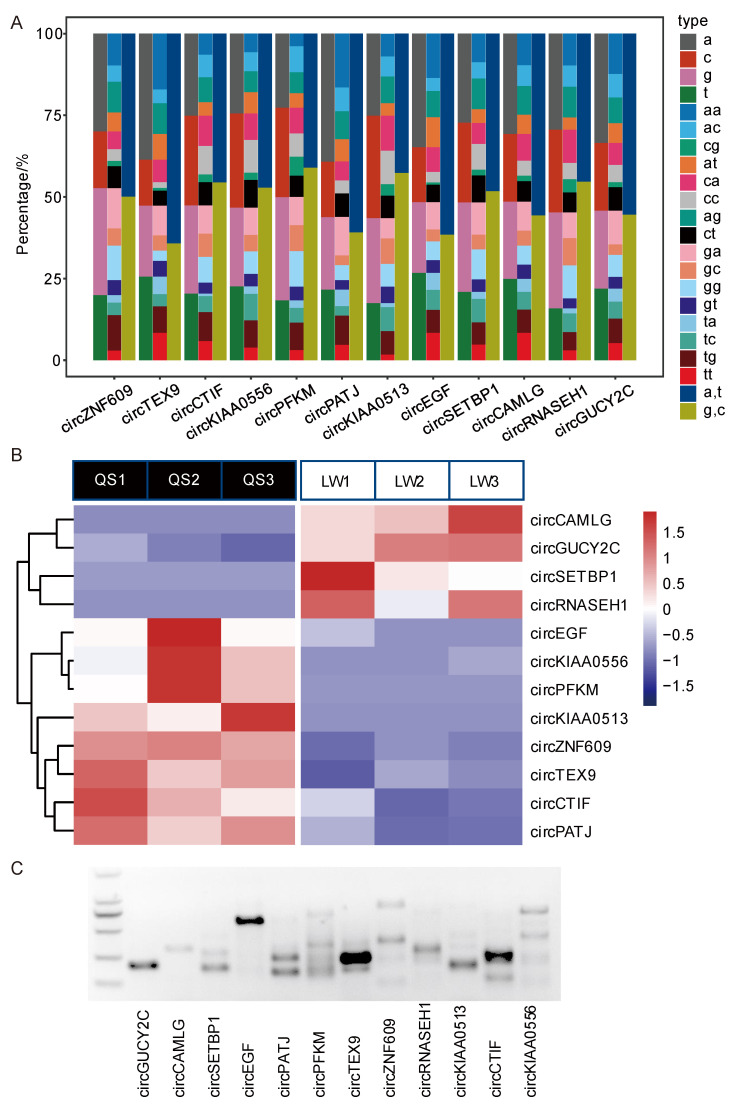
Sequence analysis and coding ability prediction of putative m6A-circRNAs. (**A**) Comprehensive analysis of the nucleotide composition of putative m6A-circRNAs and the frequency distribution of different nucleotide pairs. (**B**) Display of heat map of the sequencing expression results of 12 putative m6A-circRNAs in Queshan Black and Large White pigs, QS denotes Queshan Black pig, and LW denotes Large White pig. (**C**) Electrophoretic profile of PCR amplification products of 12 putative m6A-circRNAs.

In this study, the putative m6A-circRNA sequences and structural characteristics were analyzed. It was revealed that the contents of adenine (A) and thymine (T) were comparable to those of guanine (G) and cytosine (C). Furthermore, GA, AG, AA and TG were enriched in these 12 putative m6A-circRNAs (Figure 2A). In addition, 12 putative m6A-circRNAs showed highly significant differences in their expression profiles between Queshan Black and Large White pigs (Figure 2B). Furthermore, the results of RT-PCR uncovered the possible presence of splice variants in most of these 12 putative m6A-circRNAs (Figure 2C). Increasingly, circRNAs have been found to have the potential to encode proteins and therefore their coding ability was also predicted in this study. However, due to circRNA is structurally different from mRNA, it does not possess a 5′ cap structure. Nevertheless, the internal ribosome entry site (IRES) is an important regulatory element in RNA translation that functions independently of the 5′ cap structure, and IRES can mediate the assembly of ribosomal subunits, ultimately leading to the translation of circRNAs into peptides or proteins. Therefore, in this study, we used IRESfinder software to find whether these 12 putative m6A-circRNAs contain IRES information in their spliced sequences to predict whether they have the ability to initiate translation without relying on the 5′ cap structure. The results showed that 8 of the 12 putative m6A-circRNAs had IRES information (Table 2). It was found that some circRNAs could translate functional peptides in a cap-independent manner through small ORFs, so to predict the coding ability of these m6A-circRNAs, ORFs were also predicted. When the minimal ORF length was 30–150 nt, except for circPFKM, circPATJ and circKIAA0513, all the other nine circRNAs had varying numbers of ORFs, but when the minimal ORF length was at 300 and 600 nt, the majority of circRNAs no longer had ORFs (Table 3).

### 3.3. Construction of Regulatory Network of Putative m6A-circRNAs

**Figure 3:**
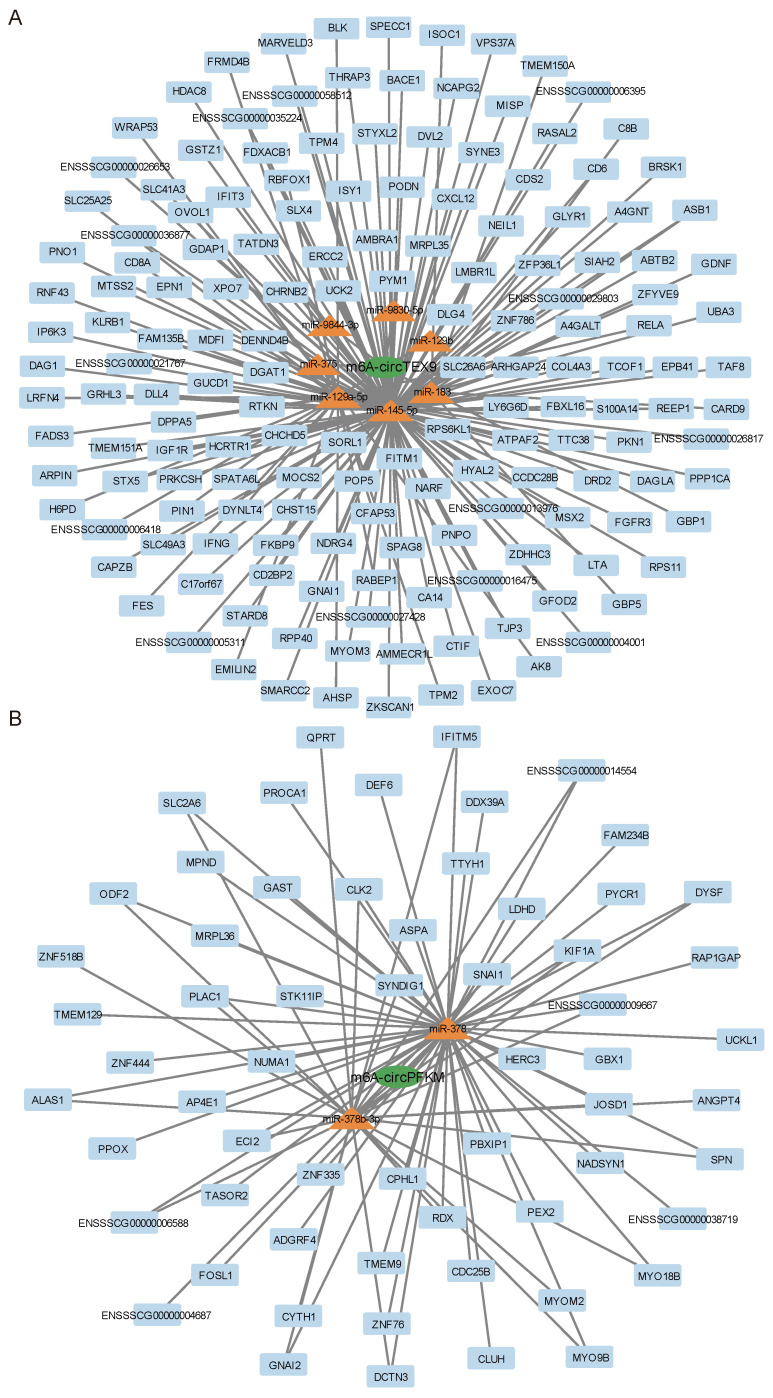

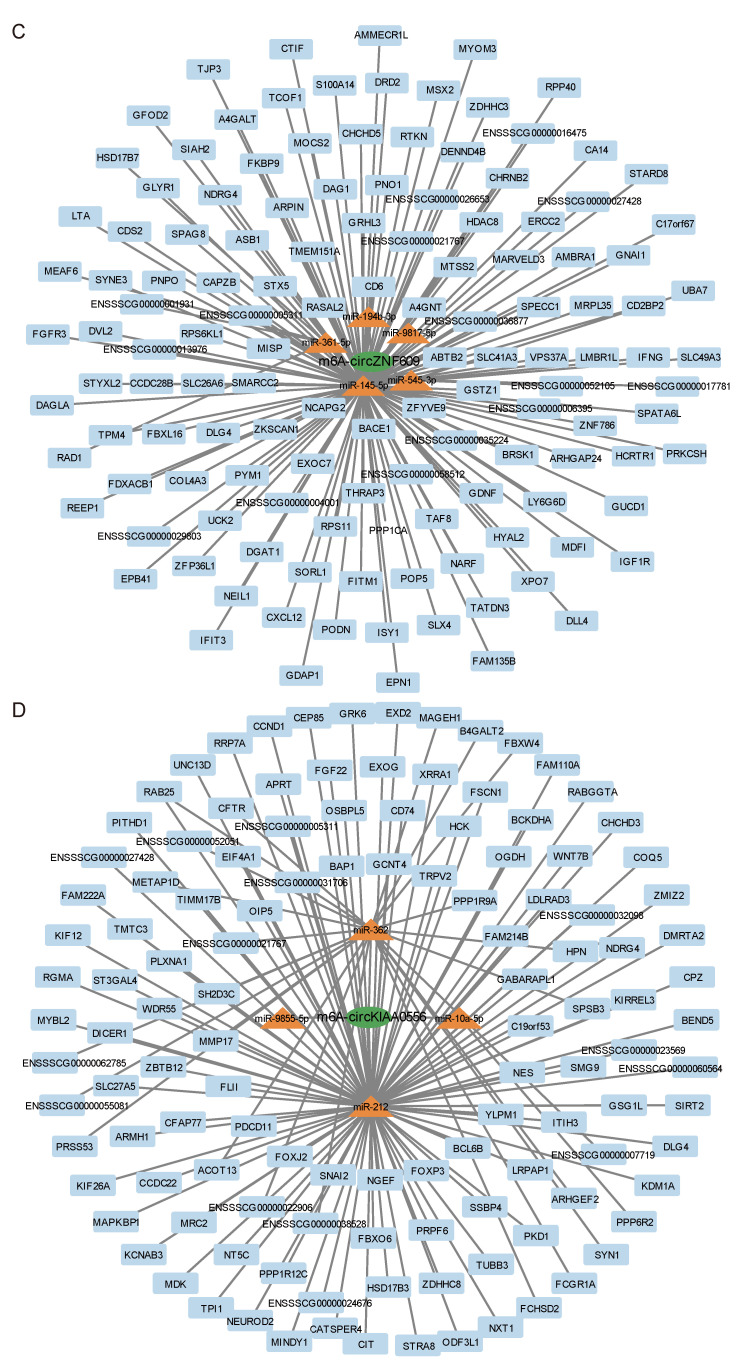
Construction of regulatory network of putative m6A-circRNAs. Construction of (**A**) m6A-circTEX9-, (**B**) m6A-circPFKM-, (**C**) m6A-circZNF609- and (**D**) m6A-circKIAA0556-related regulatory networks.

It has been increasingly found recently that m6A modifications in circRNA molecules can regulate the role of circRNAs as miRNA sponges, which in turn regulate the effects of miRNAs on target genes. To gain insight into the regulatory mechanisms of the 12 putative m6A-circRNAs obtained, this study constructed a network of competing endogenous RNAs (ceRNAs) by predicting their possible sponge miRNAs, as well as the miRNA-targeted genes. Each putative m6A-circRNA was found to sponge several miRNAs, which in turn target different genes, and the same miRNA also acts as a target for different m6A-circRNAs (Figure 3A–D, the regulatory networks of the remaining m6A-modified circRNAs are included in the Supplementary Materials). For example, m6A-circTEX9 could sponge seven miRNAs (miR-375, miR-183, miR-9844-3p, miR-9830-5p, miR-129a-5p, miR-129b, and miR-145-5p) and further regulate the expression of its target genes through the ceRNA network mechanism (Figure 3A).

### 3.4. GO and KEGG Annotation and Enrichment Analysis of Putative m6A-circRNAs

**Figure 4:**
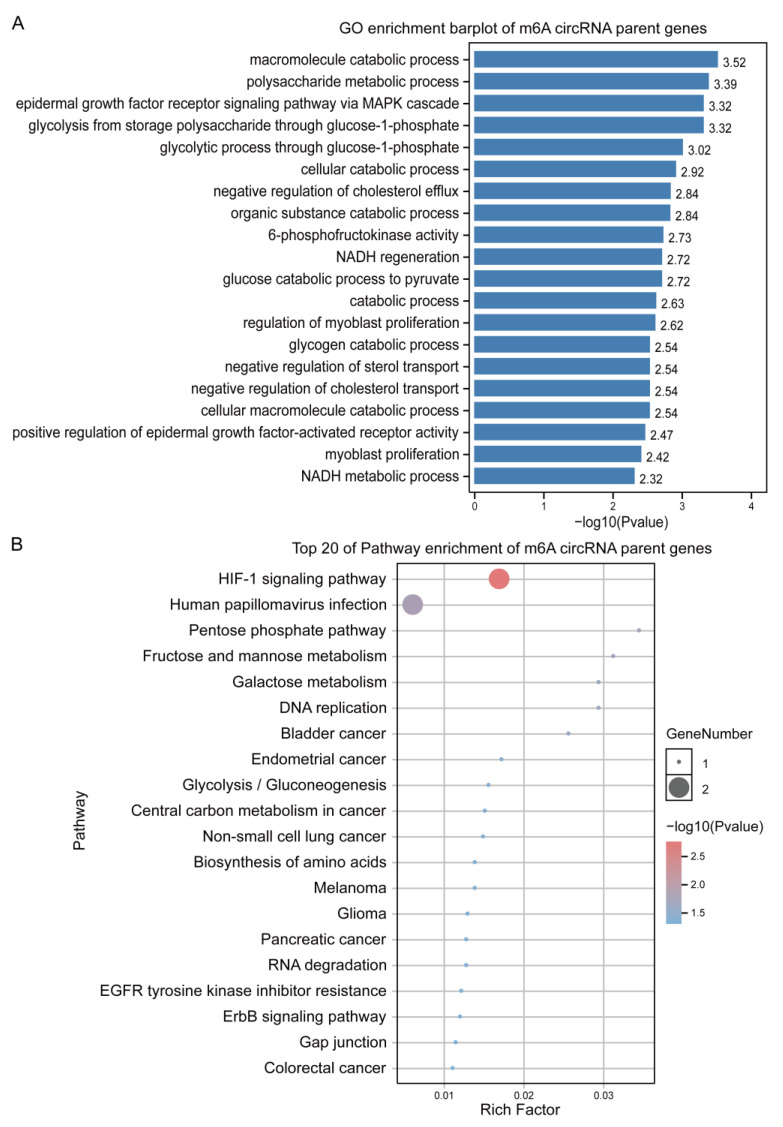
GO and KEGG annotation and enrichment analysis of putative m6A-circRNAs. (**A**) GO enrichment analysis of putative m6A-circRNAs parent genes of Queshan Black versus Large White pigs. BP: biological process; CC: cell components; MF: molecular function. (**B**) The putative m6A-circRNAs parent genes of Queshan Black versus Large White pigs were subjected to pathway enrichment analysis.

To further determine the functions of the putative m6A-circRNAs, the parent genes of these 12 putative m6A-circRNAs were functionally annotated using the GO and KEGG tools in the OmicShare online platform. The GO annotation results revealed that these genes were mainly annotated in terms such as the metabolic process and regulation of the biological process in the biological process category. The molecular functional category mainly includes binding and catalytic activity, while the cellular components mainly include cell, cell part, and organelle (Figure S1). In addition, many significantly enriched GO terms are closely associated with cellular processes, glucose and lipid metabolism, and myogenic cell proliferation, such as the macromolecule catabolic process, the polysaccharide metabolic process, the epidermal growth factor receptor signaling pathway via MAPK cascade, glycolysis from storage polysaccharide through glucose-1-phosphate, the glycolytic process through glucose-1-phosphate, and regulation of myoblast proliferation (Figure 4A).

The KEGG pathway enrichment analysis revealed that these parent genes were annotated to environmental information processing (signal transduction), human diseases (infectious diseases, cancers, drug resistance, and cancer: overview), metabolism (global and overview maps, carbohydrate metabolism, nucleotide metabolism), genetic information processing (replication and repair, folding, sorting and degradation), cellular processes (cellular community—eukaryotes, cell motility), and organismal systems (Endocrine system) (Figure S2). In addition, many significantly enriched pathways are closely related to cellular processes, lipid metabolism, and cancer, such as the HIF-1 signaling pathway, the pentose phosphate pathway, fructose and mannose metabolism, endometrial cancer, melanoma, glioma, the ErbB signaling pathway, and gap junction (Figure 4B). Therefore, these putative m6A-circRNAs may play an important role in regulating intramuscular adipogenesis and lipogenesis in pigs.

### 3.5. GO and KEGG Annotation and Enrichment Analysis of Target Genes

**Figure 5:**
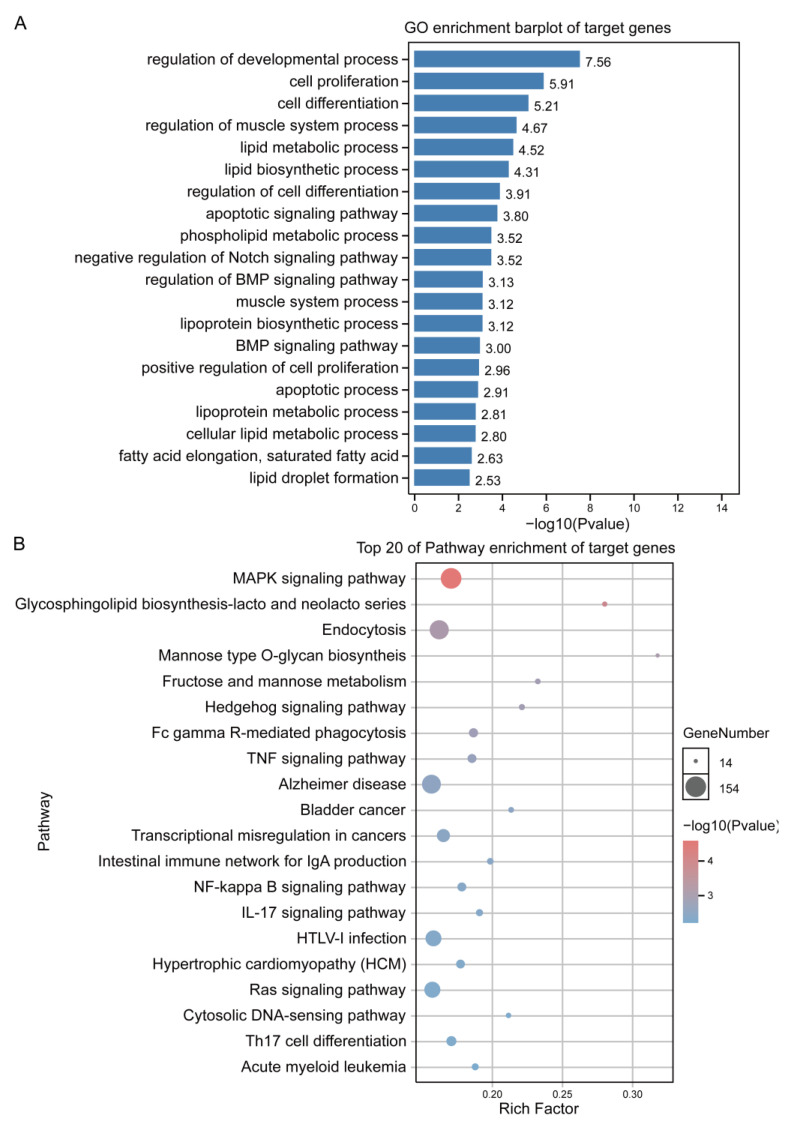
GO and KEGG annotation and enrichment analysis of target genes. (**A**) GO enrichment analysis of target genes. BP: biological process; CC: cell components; MF: molecular function. (**B**) The target genes were subjected to pathway enrichment analysis.

Because m6A-circRNA can serve as miRNA sponge through the ceRNA mechanism, and then relieve the inhibition of target genes, this study conducted functional enrichment analysis of target genes. The GO enrichment analysis showed that most of the mRNAs in the biological process category (>1000) were the cellular process, the metabolic process, biological regulation, regulation of the biological process, and response to stimulus. The majority of mRNAs in the molecular function category (>1000) were binding and catalytic activity. Based on the cellular component category, most mRNAs (>1000) were significantly enriched in cell, cell part, organelle, membrane and organelle part (Figure S3). In addition, many significantly enriched GO terms were closely related to cellular processes, cell development and lipid metabolism, such as the positive regulation of cell proliferation, the lipid metabolic process, the lipid biosynthetic process and the BMP signaling pathway (Figure 5A).

The KEGG pathway enrichment analysis revealed that these target genes were annotated to environmental information processing (signal transduction and signaling molecules and interaction), metabolism (carbohydrate metabolism, lipid metabolism), cellular processes (transport and catabolism and cellular community—eukaryotes), organismal systems (immune system and endocrine system), human diseases (infectious diseases and cancers), and genetic information processing (folding, sorting and degradation, and translation) (Figure S4). In addition, many significantly enriched KEGG pathways are closely related to cellular processes and lipid metabolism, such as the MAPK signaling pathway, the glycosphingolipid biosynthesis-lacto and neolacto series, and the TNF signaling pathway (Figure 5B).

### 3.6. Analysis of the Expression Pattern of m6A-circRNAs in the Longissimus Dorsi of Queshan Black and Large White Pigs

**Figure 6:**
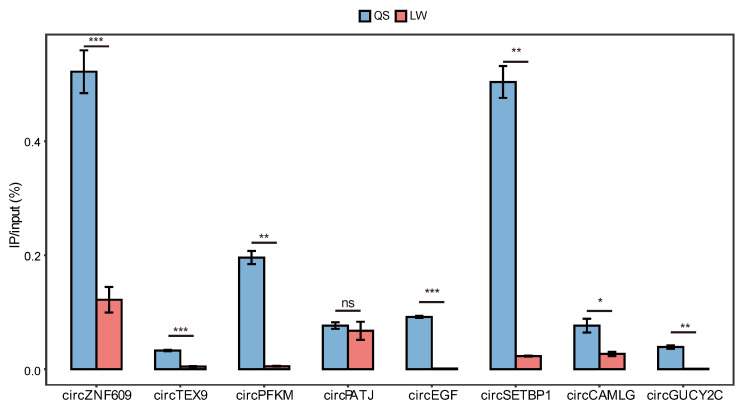
Methylation levels of eight putative m6A-circRNAs in Queshan Black and Large White pigs were detected by MeRIP-qPCR. *p* < 0.05 was considered statistically significant (* represents *p* < 0.05, ** represents *p* < 0.01, *** represents *p* < 0.001, and ns represents not significant).

In addition, we randomly selected eight putative m6A-circRNAs and analyzed their methylation in the longissimus dorsi of Queshan Black and Large White pigs using the MeRIP-qPCR technique (Figure 6). It was found that the eight analyzed m6A-circRNAs were validated in both Queshan Black and Large White pigs, and the expression of these m6A-circRNAs was higher in both Queshan Black than in Large White pigs. Many studies have shown that intramuscular fat content is higher in Queshan Black than in Large White pigs, and intramuscular fat content has an important effect on meat quality. Therefore, it is possible that these m6A-circRNAs may influence pork quality by regulating intramuscular fat deposition in pigs, and they may exert their biological functions through an m6A-dependent mechanism.

## 4. Discussion

Since circRNAs play a key role in adipogenesis and muscle development, they may have an important role in meat quality. In addition, since circRNAs are remarkably stable molecules, some circRNAs, such as chi-circ_0006511 [30], circPPARA [31] and circUBE2Q2 [32], are considered to be important targets affecting meat quality. Herein, 12 putative m6A-circRNAs containing very high confidence m6A motifs were obtained from the analysis of 64 differentially expressed circRNAs between the longissimus dorsi of Queshan Black and Large White pigs based on previous sequencing results, it is noteworthy that each motif of the m6A site in the obtained circRNA sequence is consistent with the motif of the m6A site. CircRNA is a closed-loop RNA molecule formed by post-transcriptional splicing, which plays an important role in gene expression regulation, cell proliferation, and tumor occurrence [33]. The diversity of circRNA comes from different splicing patterns of their genomic locations, which can produce different types of circRNA variants. With the continuous development of high-throughput sequencing technology, more and more circRNA splicing variants have been discovered, which helps us better understand the role of circRNA in cellular processes [34,35]. In this study, RT-PCR revealed that most of the 12 putative m6A-circRNAs may have splicing variants, but further experimental verification is needed. CircRNA splicing variants are an important aspect of circRNA research, and their discovery and analysis help us better understand the function and mechanism of these molecules.

More and more studies in recent years have found that m6A-modified circRNA molecules can serve as miRNA sponges, thus affecting the action of miRNAs on target genes. The m6A modification has been identified as the most abundant internal epistatic transcriptome modification in eukaryotic messenger RNA [36]. M6A is assembled by a “writer”, m6A methyltransferase, and the removal step is performed by m6A demethylase, also known as an “eraser”, and is recognized by specific RNA binding proteins (also known as “readers”) [37]. M6A-mediated circMDK upregulation promotes hepatocellular carcinoma progression through miR-346/874-3p-ATG16L1 axis promotes tumorigenesis and serves as a nanotherapeutic target for hepatocellular carcinoma [38]. METTL14-mediated m6A modification of circORC5 inhibits the growth and invasion of gastric cancer cells through regulation of the miR-30c-2-3p/AKT1S1 axis and may provide a promising prognostic factor for gastric cancer [39]. Although m6A modification plays a key role in a variety of cancers, little research has been done on m6A modification of circRNAs in the field of pork quality. Therefore, to further elucidate the mechanism of m6A-circRNA effects on fat deposition in pigs, we constructed an m6A-circRNA competitive endogenous RNA regulatory network based on bioinformatic analysis. It was found that each analyzed m6A-circRNA exhibited several m6A-circRNA–miRNA–mRNA regulatory pathways.

The fat deposition process in pigs is regulated by many genes involving multiple signaling pathways [40], which implies that an integrated regulatory network of related signaling pathways and regulatory genes will help to reveal the functional roles and regulatory mechanisms affecting m6A-circRNAs in pork quality. In this study, GO and KEGG enrichment analysis was performed. Interestingly, the enriched signaling pathways in m6A-circRNA parent genes are associated with lipid metabolic processes. In previous studies, it was found that the HIF-1 signaling pathway extensively regulates fat development. Foxn1 and HIF-1α (the active subunit of HIF-1, the main part of HIF-1 functions) synergistically regulate dermal white adipose tissue through the Igf2 signaling pathway during the proliferation stage of skin wound healing [41]. HIF-1α activation, FGF-2 production, and the ERK1/2 and Akt pathways are involved in the regulation mechanism of hypoxia-enhanced adipose tissue-derived stem cell proliferation [42]. The impaired wound healing ability of human adipose-derived stem cells may occur through the miR-1248/CITED2/HIF-1α pathway [43]. It was found that endogenous synthesis of erythritol by glucose via the pentose-phosphate pathway may contribute to the association between erythritol and obesity observed in young adults through in vivo conversion of erythritol to erythrophosphonate in stable isotope-assisted ex vivo blood incubation experiments [44]. In addition, inhibition of the pentose-phosphate pathway may be an early marker of adipose dysfunction in diet-induced obesity [45]. Aspirin-induced suppression of adipogenesis is p53 dependent and is associated with the inactivation of the pentose-phosphate pathway, and blocking the pentose-phosphate pathway may be a novel strategy for the prevention and treatment of obesity [46]. Significantly enriched cancer-related pathways are also associated with adipogenesis and obesity, such as Endometrial cancer [47,48], Melanoma [49], and Glioma [50]. At the same time, this study also found that some signaling pathways regulating cell proliferation and differentiation and cell communication were significantly enriched, such as fructose and mannose metabolism, the ErbB signaling pathway and Gap junction. Both fructose and mannose metabolism play an important role in glucose uptake and energy metabolism. Glycolysis/gluconeogenesis is closely related to glucose metabolism, fatty acid uptake and oxidation [51]. Interestingly, the parent genes of the putative m6A-circRNA contained in these pathways include EGF and PFKM, which numerous studies have shown to be extensively involved in lipid synthesis and catabolism. Endothelial cells are able to regulate lipolysis through the release of factors such as epidermal growth factor (EGF), which also acts as a stimulator to promote adipose stem cell proliferation, migration and invasion, and transdifferentiation into an immunophenotype of epidermal stem cells [52,53]. PFKM has been found to be not only a hub gene, but also a differentially expressed gene, which may be a strong candidate gene for influencing fat deposition in Landrace and Songliao black sows [54]. EGF and FPKM are the parent genes of m6A-circEGF and m6A-PFKM, respectively. Therefore, m6A-circEGF and m6A-PFKM may regulate lipid synthesis and catabolism through the pathways mentioned above, thus affecting the quality of pork. Importantly, the present study also found that the target genes were also significantly enriched in signaling pathways related to adipogenesis and cell proliferation and differentiation, such as the MAPK signaling pathway, the glycosphingolipid biosynthesis-lacto and neolacto series, the hedgehog signaling pathway, and the TNF signaling pathway.

Therefore, it can be speculated that genes potentially regulated by m6A-circRNAs may be involved in the physiological process of adipogenesis in pigs, and their functions may be finely regulated by m6A-circRNAs in an m6A-dependent manner, or even m6A-circRNAs act as a mechanism for miRNA sponges to regulate fat deposition.

## 5. Conclusions

In conclusion, a total of 12 putative m6A-circRNAs were identified and characterized from the longissimus dorsi of Queshan Black and Large White pigs. Eight m6A-circRNAs (m6A-circZNF609, m6A-circTEX9, m6A-circCTIF, m6A-circKIAA0556, m6A-circPFKM, m6A-circPATJ, m6A-circKIAA0513 and m6A-circEGF) were confirmed to be significantly more expressed in the longissimus dorsi of Queshan Black than in Large White pigs, while the expression of the remaining four (m6A-circSETBP1, m6A-circCAMLG, m6A-circRNASEH1 and m6A-circGUCY2C) was the opposite. The combined regulatory network and enrichment analysis indicated that they have potential functional roles on pork quality and that m6A-circRNA may affect the expression of target genes by serving as miRNA sponges, thereby regulating fat deposition.

## Figures and Tables

**Table 1:** List of primer sequences used in qPCR.

circRNA_ID	Forward Primer (5′ to 3′)	Reverse Primer (5′ to 3′)
circZNF609	GACAGTGGGGATGAATGGGA	TGTTCTCAGACCTGCCACAT
circTEX9	AAAGTGCCATGGAAGTTCGC	TTCCTCCAGTTGCCCTTCAA
circCTIF	TGATTCCTTCAGTGGTGCCA	GCTGGGCAAATCTTCAAGGT
circKIAA0556	CTGAACACACATGGATGCCC	ATCCTTGGCCTTGACACTCA
circPFKM	GGTAAGATCACAGCGGAGGA	GTTCCCGAAAGTCCTTGCAC
circPATJ	CCTATTGGGCCCTGTATGAAG	AGTTTCTGTTTCCACTGCTCC
circKIAA0513	CTCATTGGCAAAGAGACCGG	TTCTCAGCCACACTAAGGGA
circEGF	TTCCCGTGTTCTCTTAAGCG	CATCTGCCACCAATTGCTCA
circSETBP1	GGAGGAGGAAAGAGCCACT	TTCTCAGGAGGGTAAGGGA
circCAMLG	CTTGCCTTTGGAGTCAGAGC	CCGAATCACCCAGCACTACT
circRNASEH1	GGAGGTGGTCAACAAAGAGG	CGTGTAGAGAACAGCTTGC
circGUCY2C	TCTGGTGGAGGAAAGGACAC	TTTTGGTCATGGAAGAGGCC

**Table 2:** Analysis of 12 putative m6A-circRNAs sequences with IRES.

circRNA ID	Index	Score
circZNF609	non-IRES	0.342887
circTEX9	IRES	0.561539
circSETBP1	IRES	0.522931
circCTIF	IRES	0.560397
circCAMLG	IRES	0.660322
circRNASEH1	non-IRES	0.190536
circKIAA0556	IRES	0.529494
circGUCY2C	IRES	0.594912
circPFKM	non-IRES	0.141979
circPATJ	IRES	0.582689
circKIAA0513	non-IRES	0.264454
circEGF	IRES	0.804553

**Table 3:** ORF prediction statistics for 12 putative m6A-circRNAs.

circRNA_ID	Minimal ORF Length (nt)				
	30	75	150	300	600
circZNF609	5	2	1	0	0
circTEX9	8	4	1	0	0
circCTIF	5	3	3	0	0
circKIAA0556	5	4	1	1	1
circPFKM	3	2	0	0	0
circPATJ	3	1	0	0	0
circKIAA0513	4	3	0	0	0
circEGF	5	2	1	1	0
circSETBP1	3	3	1	1	0
circCAMLG	4	3	3	2	1
circRNASEH1	2	2	2	1	0
circGUCY2C	2	1	1	0	0

## Data Availability

Not applicable.

## Supplementary Materials

The following supporting information can be downloaded at: https://www.mdpi.com/article/10.3390/ani13132190/s1, Figure S1: GO secondary classification of m6A-circRNA parent genes; Figure S2: KEGG pathway annotation of m6A-circRNA parent genes; Figure S3: GO secondary classification of target genes; Figure S4: KEGG pathway annotation of target genes; Table S1: List of m6A-circCAMLG-related ceRNA regulatory networks; Table S2: List of m6A-circCTIF-related ceRNA regulatory networks; Table S3: List of m6A-circEGF-related ceRNA regulatory networks; Table S4: List of m6A-circGUCY2C-related ceRNA regulatory networks; Table S5: List of m6A-circKIAA0513-related ceRNA regulatory networks; Table S6: List of m6A-circPATJ-related ceRNA regulatory networks.xlsx; Table S7: List of m6A-circRNASEH1-related ceRNA regulatory networks; Table S8: List of m6A-circSETBP1-related ceRNA regulatory networks.
