# Supplementary material for: Expression Profile and Regulatory Properties of m6A-Modified circRNAs in the Longissimus Dorsi of Queshan Black and Large White Pigs
Source: Animals (Basel). 2023 Jul 4;13(13):2190. doi: 10.3390/ani13132190 (PMC10339870; doi:10.3390/ani13132190)
Supplement: Supplementary file 1 [file animals-13-02190-s001.zip › Supplementary Files/animals-2387305-supplementary.pdf]

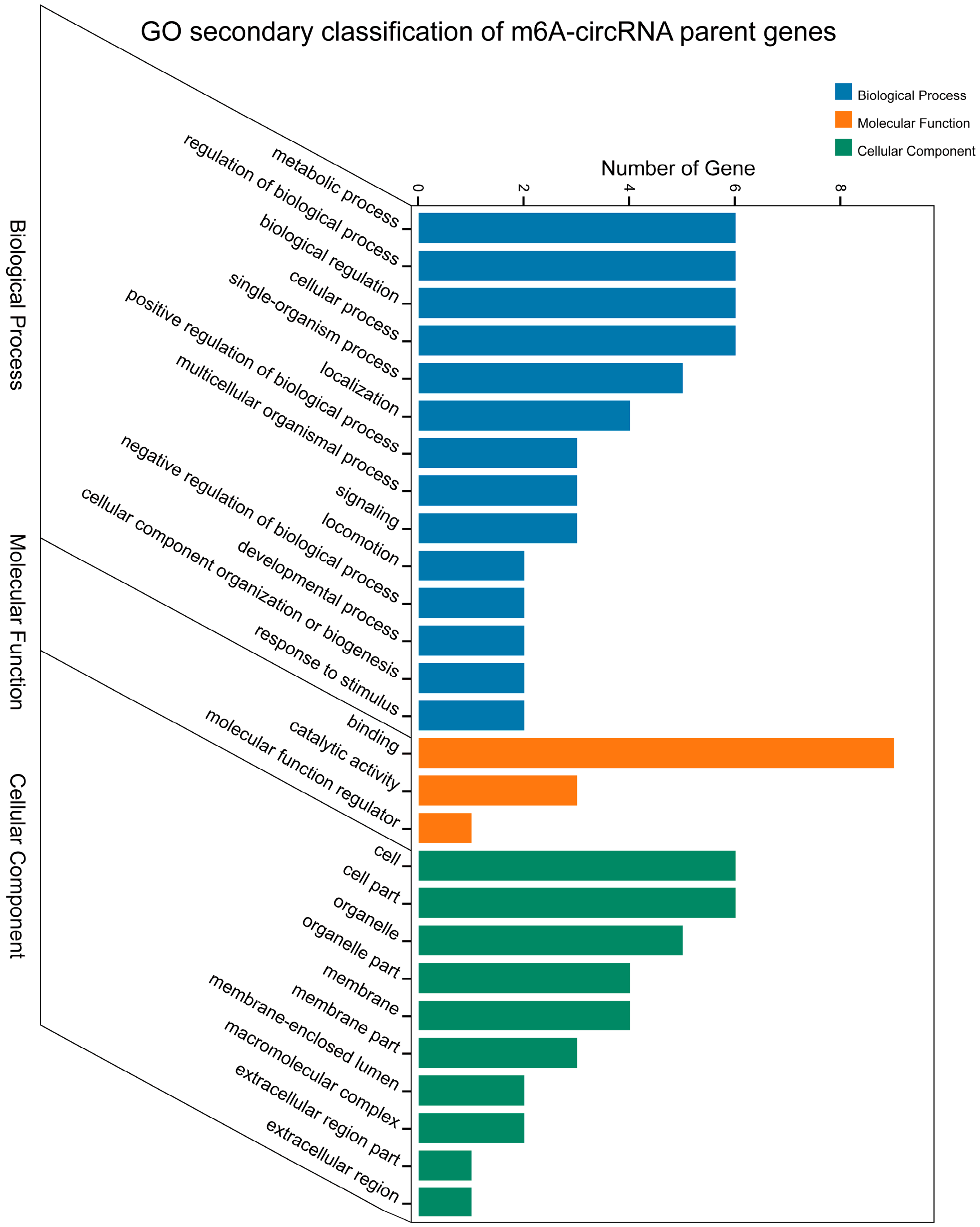

Figure S1. GO secondary classification of m6A-circRNA parent genes.

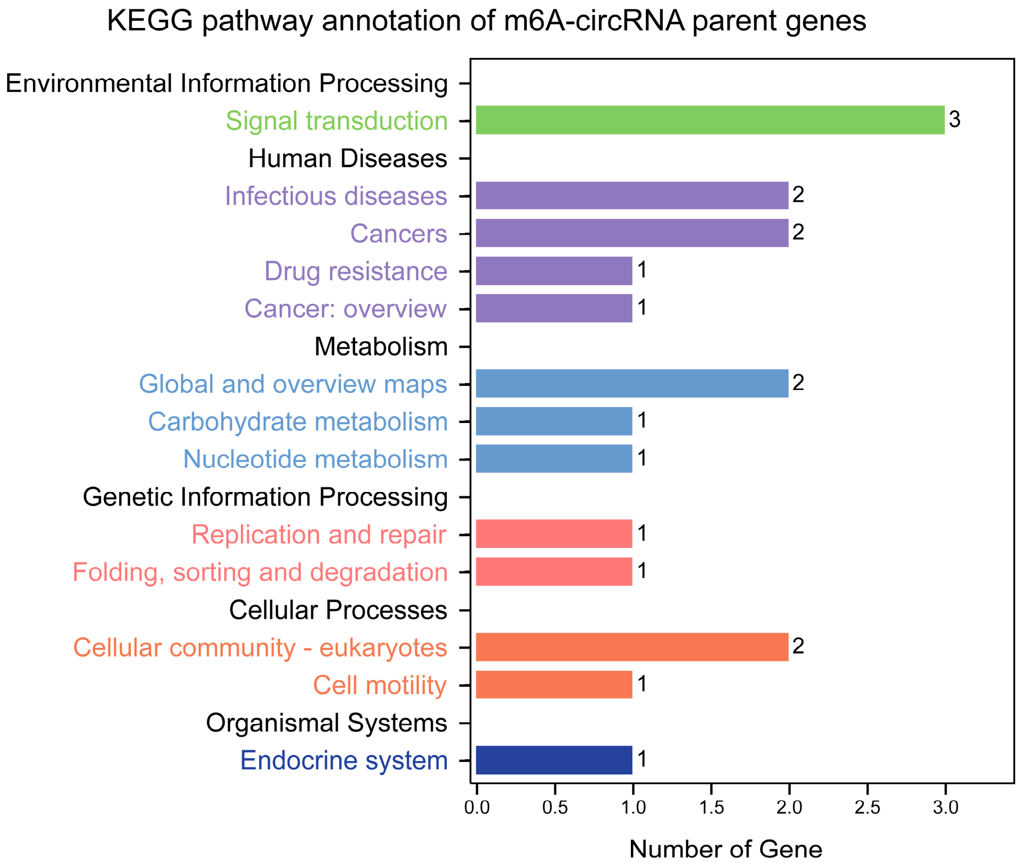

Figure S2. KEGG pathway annotation of m6A-circRNA parent genes.

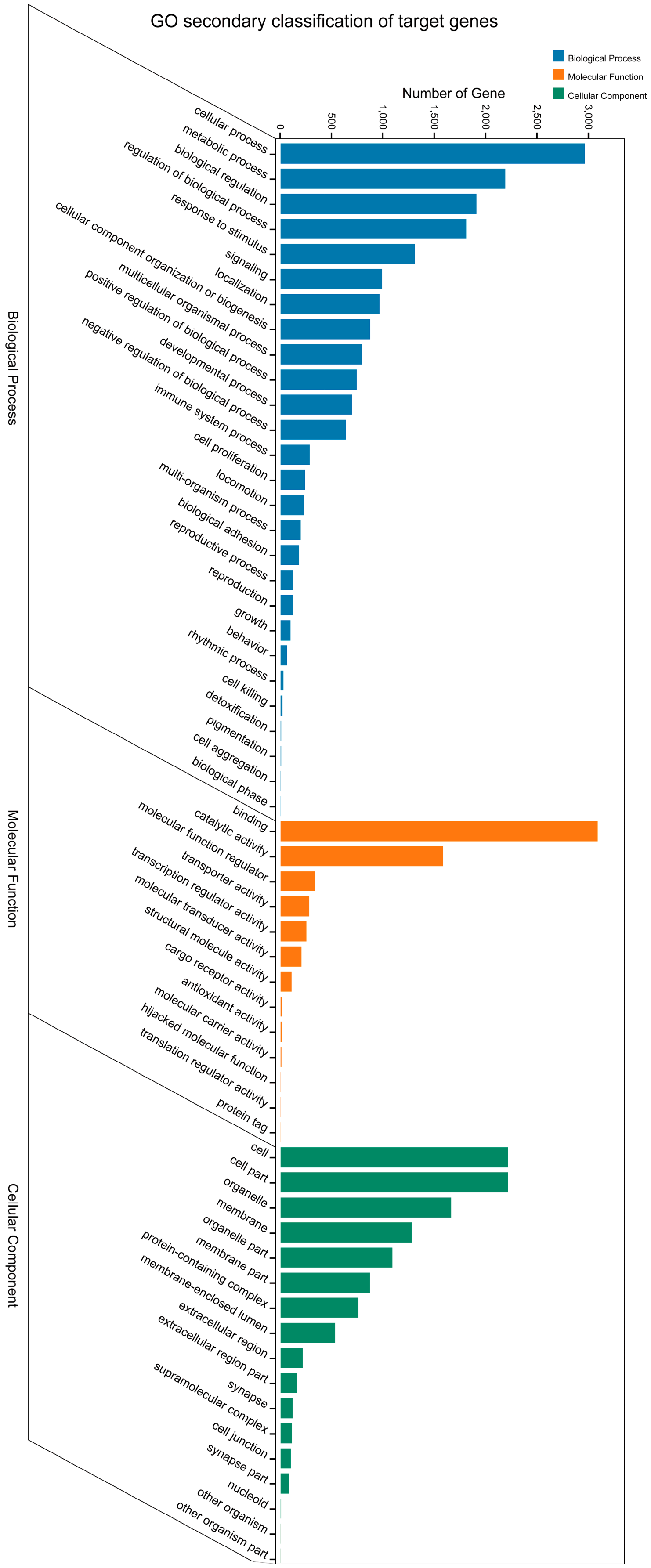

Figure S3. GO secondary classification of target genes.

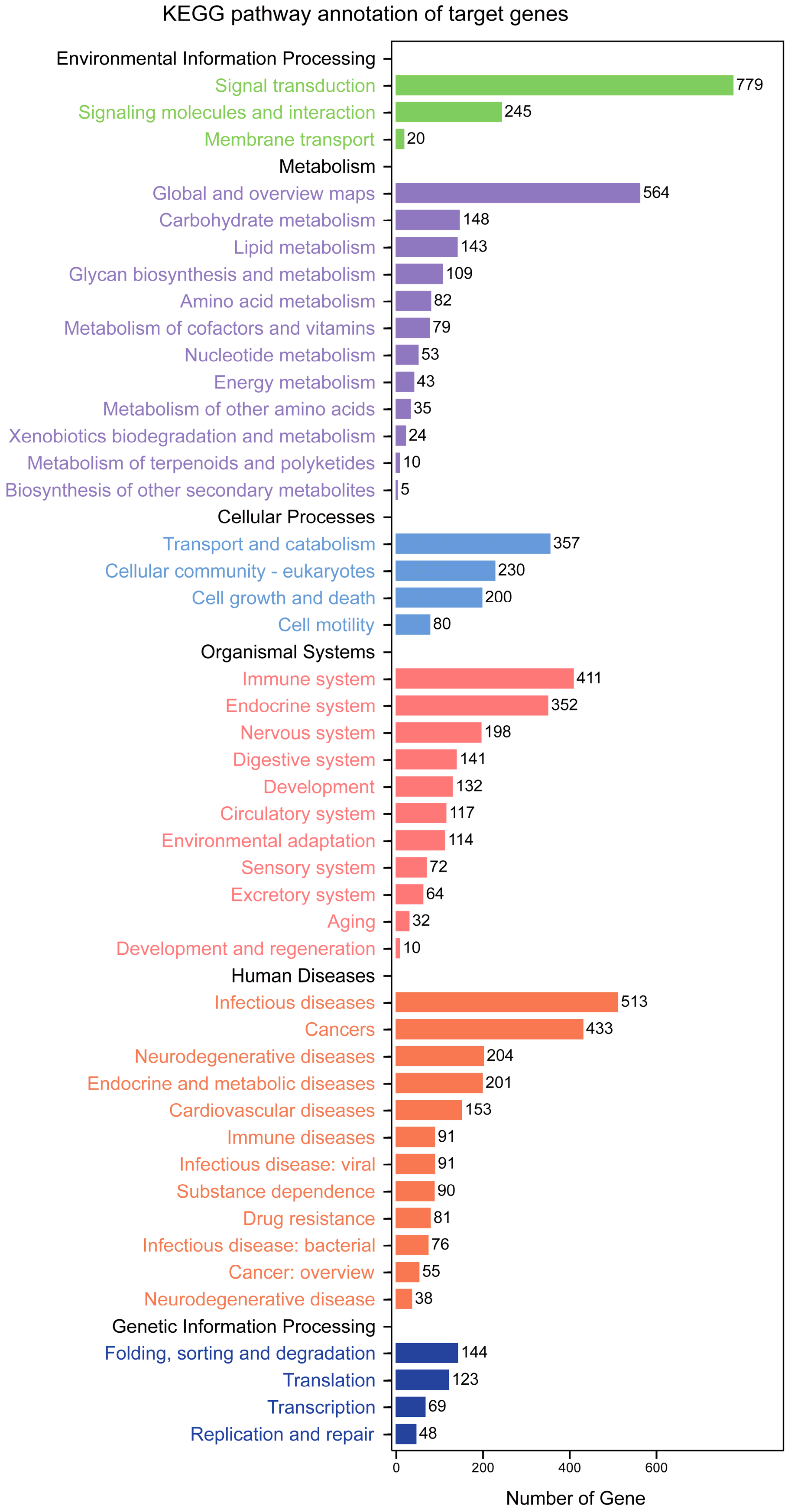

Figure S4. KEGG pathway annotation of target genes.
